# Regulation of focal adhesion turnover in SDF-1α-stimulated migration of mesenchymal stem cells in neural differentiation

**DOI:** 10.1038/s41598-017-09736-7

**Published:** 2017-08-30

**Authors:** Ya’nan Hu, Junhou Lu, Xiaojing Xu, Jingya Lyu, Huanxiang Zhang

**Affiliations:** Department of Cell Biology, Jiangsu Key Laboratory of Stem Cell Research, Medical College of Soochow University, Suzhou, 215123 China

## Abstract

Directed migration of the transplanted mesenchymal stem cells (MSCs) to the lesion sites plays a pivotal role in the efficacy of cell-based therapy. Our previous study demonstrates that MSCs under varying neural differentiation states possess different migratory capacities in response to chemoattractants. However, the underlying mechanism has not been fully addressed. Herein, we show that the assembly and turnover of focal adhesions, the phosphorylation of FAK and paxillin, and the reorganisation of F-actin in MSCs are closely related to their differentiation states in response to SDF-1α. Upon SDF-1α stimulation, FAs turnover more rapidly with the most obvious reduction in the existing time of FAs in MSCs of 24-h preinduction that exhibit the most effective migration towards SDF-1α. Further, we confirm that PI3K/Akt and MAPK pathways participate in the regulation of SDF-1α-induced cell migration and FA assembly, and moreover, that the regulatory effects vary greatly depending on the differentiation states. Collectively, these results demonstrate that FA assembly and turnover, which is accompanied with F-actin reorganisation in response to SDF-1α, correlates closely with the differentiation states of MSCs, which might contribute to the different chemotactic responses of these cells, and thus help develop new strategy to improve the efficacy of MSCs-based therapy.

## Introduction

Mesenchymal stem cells (MSCs), a widely-studied adult stem cell population, are characterised by high proliferative and differentiation abilities, low immunogenicity, neuroprotective effects and potential of neural differentiation^1^, highlighting the clinical applicability of these cells. However, studies have shown that only a small percentage of the transplanted MSCs can reach the lesions, leading to a very low rate of cell replacement^2^. Thus, enhancing the migratory capacity of MSCs is critical to maximize the effectiveness of MSCs-based therapy.

Many growth factors and cytokines have been found to act as strong chemoattractants for MSCs^3^, among which stromal cell-derived factor-1α (SDF-1α, also known as CXCL12) has received much attention^4^. SDF-1α through its cognate receptor CXC chemokine receptor 4 (CXCR4), plays a pivotal role in migration, engraftment and survival of MSCs, and stimulates the homing of transplanted MSCs to various target organs including damaged brain^5–8^.

Cell migration is a complex process that results from the ordered changes in the cytoskeletons and the regulated formation, turnover and distribution of focal adhesions (FAs)^9^. FAs lie at the convergence of extracellular matrix on the outside, integrin signalling and actin cytoskeleton on the inside, which consist of a series of structural and signalling molecules such as integrin, focal adhesion kinase (FAK), vinculin, and paxillin^10, 11^. In fast migrating cells, lamellipodium, one of the protruding cellular structures, covers the front of the cell like a crescent, which continuously advances due to assembly of smaller FAs, which are typically less than 2 μm in length, and polymerisation of F-actin at the leading edge, while actin filaments are bundled into stress fibers anchored with larger FAs located at the cell periphery in slow migrating cells^12, 13^. Studies have shown that tyrosine phosphorylation of focal adhesion proteins, including FAK on Y397, paxillin on Y31 and Y118 is increased in migrating cells^14, 15^ and that FA signalling plays an important role in SDF-1α-induced cell migration^16, 17^. Moreover, stimulation of MSCs with SDF-1α significantly results in the activation of FAK and paxillin, and the rearrangement of F-actin^18^.

It is widely studied that phosphatidylinositol 3-kinase (PI3K) and mitogen-activated protein kinase (MAPK) signalling pathways are involved in the regulation of the directed migration of many kinds of cells^19, 20^, and FA signalling activation and cytoskeletal reorganisation that are essential for cell migration^21–23^. Our previous study showed that there is a close relationship between the chemotactic responses of MSCs or neural stem cells and their differentiation states, and that cells at a certain level of differentiation can be endowed with stronger migratory capacity and display much more potent chemotactic responsiveness^24–26^. We speculate that the formation and turnover of FAs, as well as the rearrangement of F-actin correlate closely with the differentiation states, and further that these differences could contribute to the variation of chemotactic responses among the differentiating MSCs.

To test this hypothesis, we induced neural differentiation of MSCs *in vitro* and analysed the turnover of FAs during chemotactic responses of these cells to SDF-1α in relation to their differentiation states. We demonstrate that the assembly and turnover of FAs, activation of FAK and paxillin, and organisation of F-actin in MSCs are closely related to their differentiation states during the chemotactic migration towards SDF-1α, and that PI3K/Akt and/or MAPK signalling pathways participate in the regulation of these events with different degrees, thus contributing to the varying chemotactic responses of the differentiating MSCs.

## Results

**Figure 1:**
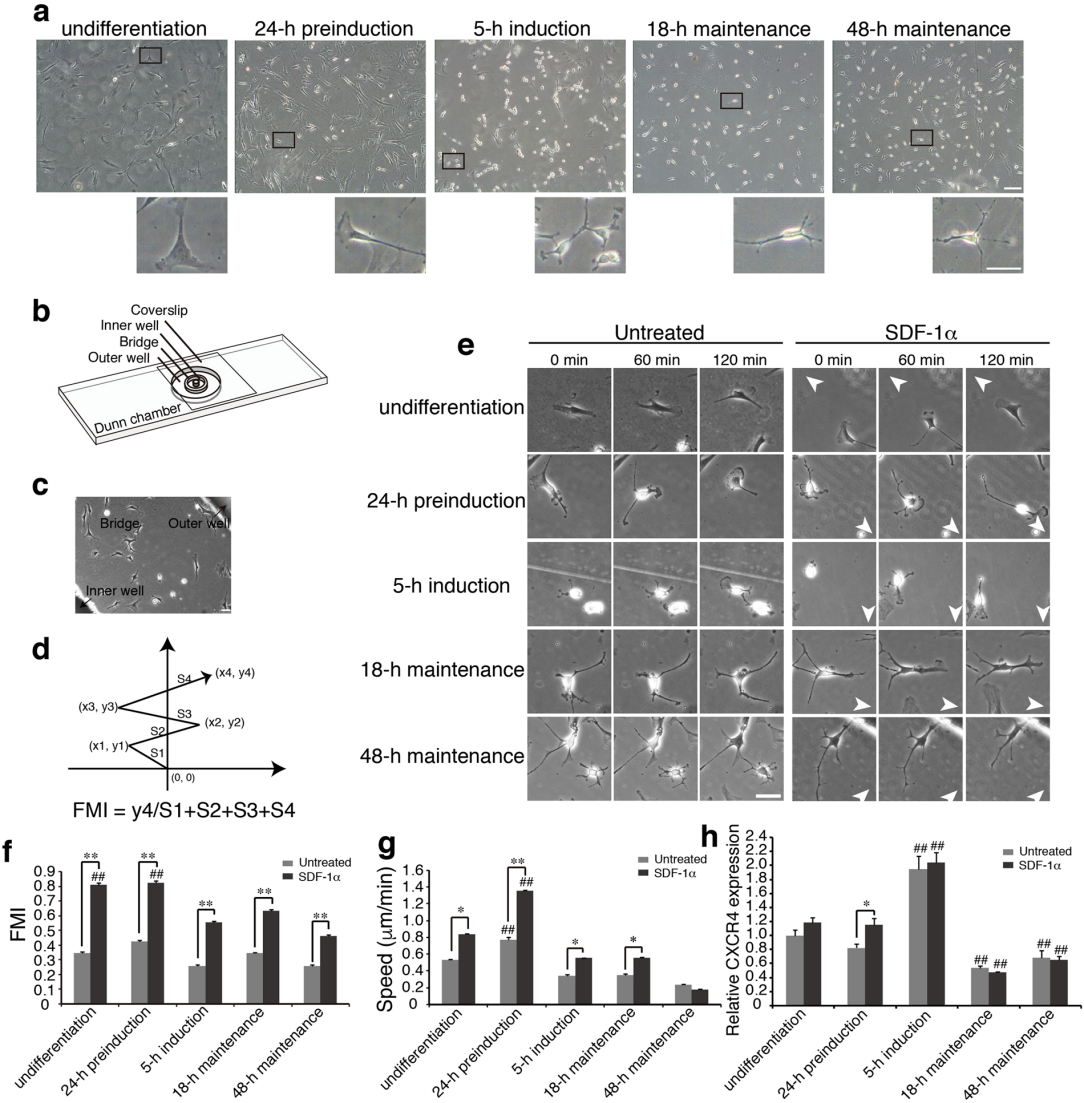
Migration tracks of cells under varying neural differentiation states in response to SDF-1α. (**a**) Morphological features of MSCs under varying neural differentiation states including undifferentiation, 24-h preinduction, 5-h induction, 18- and 48-h maintenance (upper panel) and the representative cell of each state in the boxed area is enlarged to the bottom right corner (lower panel). (**b**) Schematic representation of Dunn chamber with the overlying coverslip, showing the position of the inner well, bridge, and outer well. (**c**) Cells over the annular bridge between the inner and outer well of Dunn chamber can be observed under phase-contrast optics. The black arrow on the top right corner indicates the direction of the outer well and the left bottom one indicates the inner well. Cell migration on the bridge was recorded continuously by time-lapse frame grabbing. (**d**) Schematic diagram of migration tracks, the starting point for each cell is the intersection between the X- and Y- axes (0, 0), and the source of SDF-1α is at the top. (**e**) Representative time-lapse sequences of cell migration in varying neural differentiation states in the absence or presence of SDF-1α. Arrowheads indicate the source of SDF-1α. (**f**–**h**) FMI values, migration speed (μm/min) and CXCR4 expression of MSCs under varying differentiation states. FMI is the ratio of effective displacement to the total path length^28^. Migration speed was calculated for each time-lapse interval (5 minutes), and the mean speed was derived for 4 hours. CXCR4 expression was detected by RT-qPCR. Untreated cells were in serum-free medium without SDF-1α (bars in gray), chemotaxis was studied by adding SDF-1α in the outer well (bars in dark) (>20 cells, n = 3). Data represent the mean ± SEM from at least three independent experiments. Scale bar: 50 μm. Differences were considered statistically significant when *P < 0.05, **P < 0.01 compared with SDF-1α group of each state and ^#^P < 0.05, ^##^P < 0.01 compared with any other groups under the same treatment.

The isolation, purification and cultures as well as the characterisation and the multi-lineage differentiation, including neural differentiation of MSCs were performed as described previously^24^. Undifferentiated MSCs, grown in serum-containing L-DMEM, were fibroblast-like or long spindle-shaped. These cells were positive for CD29, CD90 and CD106, negative for CD34 and CD45, and could give rise to adipocytes and osteoblasts under appropriate differentiation conditions^24^. Upon neural differentiation, during which the expression of β-III tubulin was increased while nestin was decreased over time^24^, cytoplasm retracted towards the nucleus, cell bodies became more spherical, and process-like extensions kept elaborating. After 3 days, cells (48-h maintenance) displayed a small, round cell body with long extensions (Fig. 1a).

When switched to serum-containing medium, these differentiating cells, including MSCs in 24-h preinduction, 5-h induction, and 18- and 48-h maintenance states, not only had morphologically changed to undifferentiated MSCs but also had the antigenic phenotypes as well, and moreover could be differentiated into adipocytes, osteoblasts and neuron-like cells^24^, demonstrating that these differentiating cells are just in the early neural differentiation states.

### Chemotactic migration of MSCs in varying differentiation states towards SDF-1α

In our previously published study^26^, we demonstrated that MSCs at early neural differentiation states possess different transfilter migration and MSCs of 24-h preinduction display the strongest chemotactic response to SDF-1α by using Boyden chamber. However, it was impossible, in those experiments, to visualize the migratory behavior of MSCs that were responding to SDF-1α, and to detail the migratory responses, including migration speed and efficiency, as well as the assembly and turnover of focal adhesions (FAs). To assess the migratory behaviour of MSCs at varying neural differentiation states under the stimulation of SDF-1α, we took advantage of the direct-viewing Dunn chemotaxis chamber, which allows one to determine the direction of migration in relation to the direction of the chemoattractants’ gradient. Chemoattractants added to the outer well of the chamber can diffuse across the bridge to the inner well and form a linear steady gradient within 30 min of setting up the apparatus (Fig. 1b and c)^27^. To study the chemotaxis of the differentiating MSCs, the outer well was filled with induction medium containing SDF-1α and the concentric inner well with induction medium only, and cells cultured on the coverslip that was inverted onto the chamber were captured every 5 min for 4 h. To measure the efficiency of directed cell migration, forward migration index (FMI), the ratio of the most direct distance the cell progressed towards the gradient source to its total path length^28^, was calculated (Fig. 1d). As shown in Fig. 1e, MSCs exposed to the SDF-1α gradient displayed strong positive chemotaxis, forming large lamellipodium at the leading edge, while the unstimulated MSCs made random turns during migration. Quantitative analyses revealed that SDF-1α stimulation resulted in a significant increase in FMI, indicating the higher migration efficiency of the stimulated MSCs, with the highest migration efficiency being observed in undifferentiated MSCs and MSCs of 24-h preinduction (Fig. 1f). The migration speed of MSCs except for the cells of 48-h maintenance was also increased in response to SDF-1α, and notably the highest speed was observed in cells of 24-h preinduction (Fig. 1g). Immunocytochemical staining revealed that neither the cell morphology nor neural-like specific markers expression was changed upon SDF-1α treatment for 4 h (Fig. S1), suggesting that SDF-1α does not affect the differentiation of MSCs during the 4-h migration assay.

It has previously been reported that SDF-1α-regulated epithelial ovarian cancer cell invasion is accompanied by the increased expression of CXCR4^29^. We then examined the expression of the CXCR4 in MSCs under varying neural differentiation states upon SDF-1α treatment. RT-PCR analysis showed that MSCs in all differentiation states expressed CXCR4 with the most pronounced expression in MSCs of 5-h induction, and notably, that SDF-1α treatment resulted in a significant increase of CXCR4 expression only in MSCs of 24-h preinduction (Fig. 1h).

Taken together, these data reveal that in response to SDF-1α stimulation, the migration speed and migration efficiency of MSCs vary greatly, depending on their neural differentiation states and that MSCs of 24-h preinduction exhibit the strongest chemotactic response as compared with cells in other differentiation states.

### The assembly and size of FAs are closely related to the differentiation states of MSCs

The dynamic remodeling of FAs and the spatiotemporal rearrangement of cytoskeleton are implicated in the regulation of cell migration^30, 31^. Since MSCs of varying differentiation states have different migratory response to SDF-1α, we test whether the assembly of FAs and organisation of F-actin vary in SDF-1α-stimulated MSCs and the relationship with the differentiation of these cells. Cells were double stained with paxillin indicating FAs and TRITC-phalloidin for F-actin. There is evidence showing that fast-moving cells usually possess abundant small dot-like nascent FAs and polymerised actin at the ruffles, whereas sparse, large streak-like FAs at the periphery characterise slow-moving cells^32^. Therefore, the number and the size of FAs, which provide an assessment of the assembly of FAs was then analysed.

**Figure 2:**
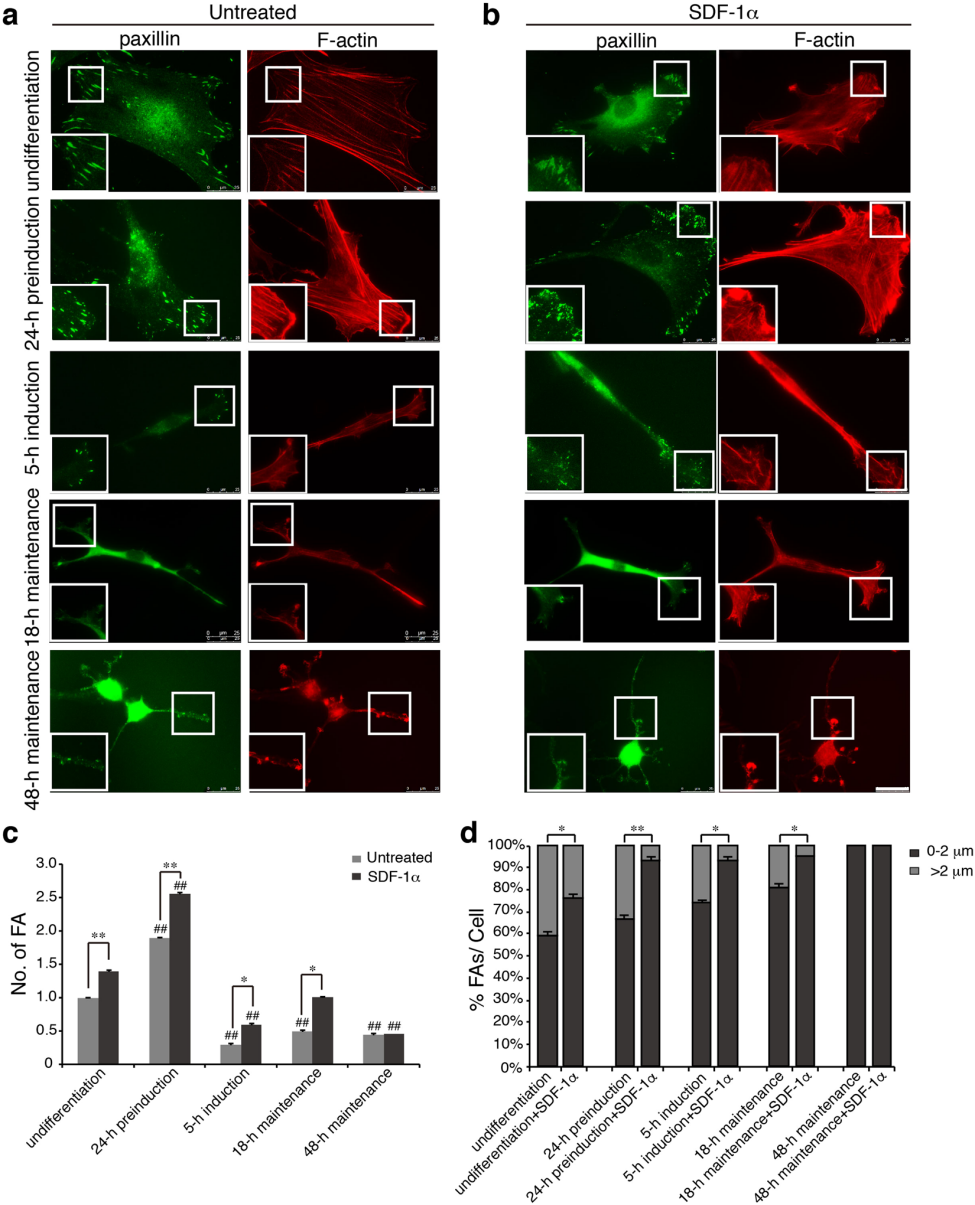
Assembly of FAs and organisation of F-actin in SDF-1α-stimulated MSCs. (**a**,**b**) MSCs treated with SDF-1α after serum-starvation were double stained with paxillin (green) and TRITC-phalloidin. The representative position in the boxed area is enlarged to the bottom left corner. Scale bar: 25 μm. (**c**,**d**) The number and length of FAs (n > 400) in untreated or SDF-1α-stimulated MSCs were measured using the NIH imageJ software. Then, the percentage of FAs (0–2 μm or >2 μm) to the total FAs (% FAs/cell) was calculated (>20 cells). Values were expressed as a percentage of undifferentiated MSCs in serum-free conditions without SDF-1α. Data represent the mean ± SEM from at least three independent experiments. Differences were considered statistically significant when *P < 0.05, **P < 0.01 compared with SDF-1α group of each state and ^#^P < 0.05, ^##^P < 0.01 compared with any other groups under the same treatment.

As shown in Fig. 2a, significant differences in the total number and size of FAs and polymerisation of F-actin were observed among MSCs of varying neural differentiation states. In the undifferentiated cells, FAs were mostly fibrillar-like and mainly localized with the tips of F-actin at the cell periphery. After preinduction for 24 h, cells displayed F-actin enriched cellular protrusions in which numerous small dot-like FAs were observed, and both the total number of FAs and the percentage of small FAs (less than 2 μm in length) were obviously increased, whereas in cells of 5-h induction, 18- and 48-h maintenance, due to, at least in part, the dramatic changes of cell morphology, the total number of FAs, mostly small dot-like, was decreased.

After treatment for 30 min by SDF-1α at 25 ng/ml, a concentration and treatment time period that induced the strongest response compared with other conditions from our preliminary experiments, MSCs exhibited a more polarized morphology, with obvious extension of membrane protrusions at the leading edge (Fig. 2b). Immunohistochemical staining of paxillin showed that the number of FAs in SDF-1α-treated MSCs was significantly increased, and small FAs appeared in higher frequency. Meanwhile, TRITC-phalloidin staining indicated that SDF-1α induced F-actin polymerisation at the leading edge revealed by the significant increase of barbed ends and elongation of F-actin (Figs S2 and S3).

Upon the stimulation of SDF-1α, except for cells of 48-h maintenance state, MSCs, especially those of undifferentiation and 24-h preinduction, were induced to reorganise the actin cytoskeleton, forming obvious broad lamellipodium at the leading edge of the migrating cells (boxed area in Fig. 2b). These structures were composed of numerous polymerised F-actin and stabilized by small FAs at their barbed ends (Fig. 2b). As quantified in Fig. 2c, SDF-1α significantly promoted the formation of FAs in cells of undifferentiation, 24-h preinduction, 5-h induction, and 18-h maintenance, but not in cells of 48-h maintenance, as compared with the unstimulated cells of the same differentiation status. The ratio of small FAs in MSCs of various differentiation states except in cells of 48-h maintenance was significantly increased by SDF-1α treatment, especially in 24-h preinduction state (Fig. 2d). Together, these results indicate that the assembly of FAs correlates closely with the differentiation of MSCs upon SDF-1α treatment, and that cells of 24-h preinduction show the most increased degree of FA assembly and ratio of small FAs that usually characterise the fast moving cells, which is consistent with the above-mentioned observations that MSCs of 24-h preinduction exhibit the most effective migration toward SDF-1α.

### Phosphorylation of FAK and paxillin in SDF-1α-stimulated MSCs of varying differentiation states

Initiating the focal adhesion signalling requires a series of tyrosine phosphorylation on adhesion molecules^33, 34^, among which the tyrosine phosphorylation of FAK on Y397 and paxillin on Y31/Y118 have been shown to be important for the assembly and turnover of FAs^35, 36^. We then examined the phosphorylation of FAK and paxillin in MSCs exposed to SDF-1α at 25 ng/ml for 30 min, a condition that relative high tyrosine phosphorylation level of these molecules was observed based on our preliminary experiments in which different concentrations (0, 5, 25, 50, and 100 ng/ml) and different times (0, 1, 5, 15, 30, and 60 min) were tested (Fig. S4).

**Figure 3:**
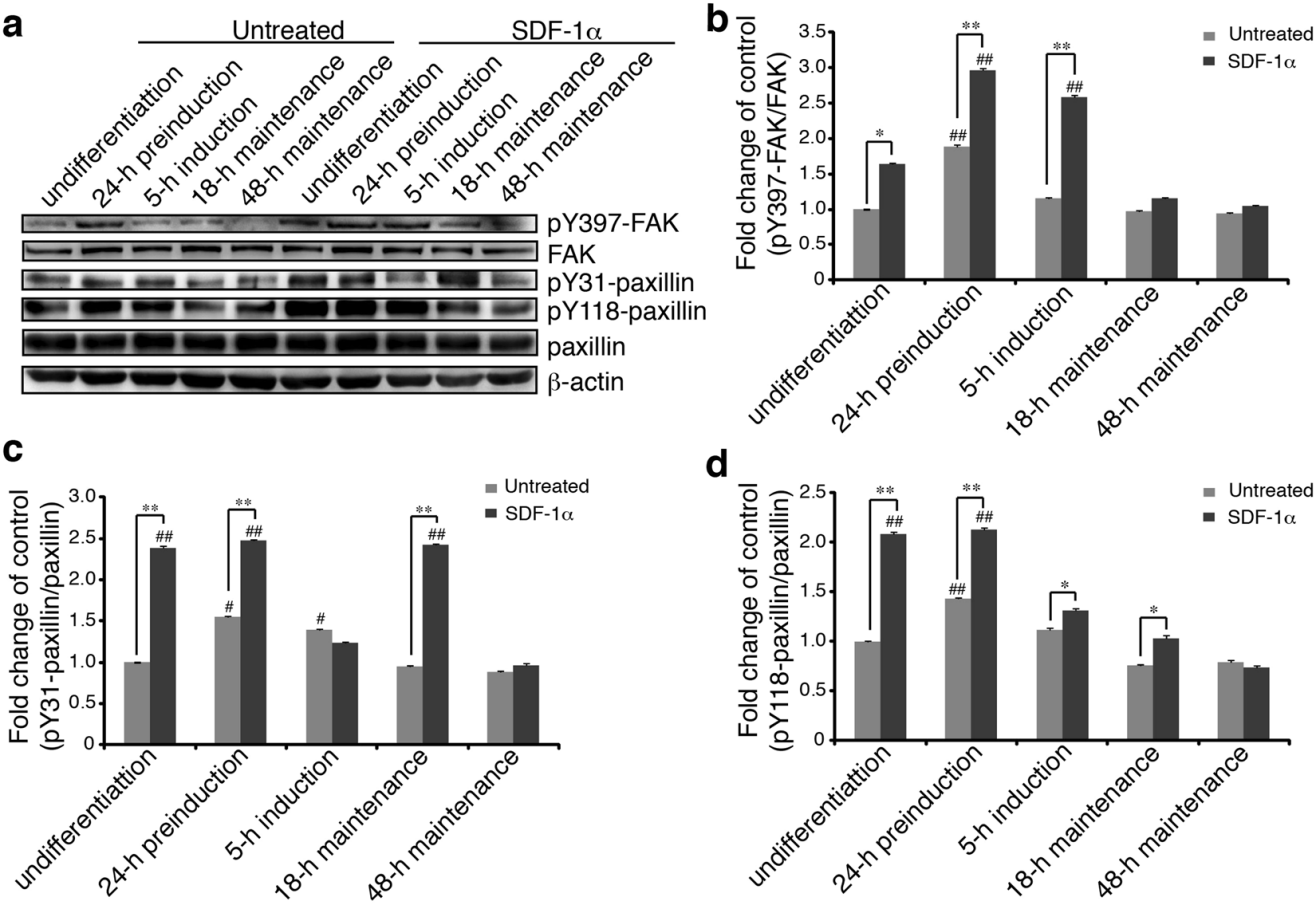
Phosphorylation of FAK and paxillin in SDF-1α-stimulated MSCs. (**a**) MSCs treated with SDF-1α after serum-starvation, were lysed and immunobloted with primary antibodies against the active phosphorylated forms of FAK and paxillin (pY397-FAK, pY31-paxillin, and pY118-paxillin) or against the total proteins (FAK and paxillin). (**b**–**d**) Levels of Y397-FAK/FAK, Y31-paxillin/paxillin and Y118-paxillin/paxillin were measured by densitometry of immunoreactive bands using ImageJ software. Values were expressed as a percentage of undifferentiated MSCs without SDF-1α treatment. Data represent the mean ± SEM from at least three independent experiments. Differences were considered statistically significant when *P < 0.05, **P < 0.01 compared with SDF-1α group of each state and ^#^P < 0.05, ^##^P < 0.01 compared with any other groups under the same treatment.

As shown in Fig. 3, a basal level of FAK and paxillin phosphorylation was observed in the undifferentiated and differentiating MSCs. Upon SDF-1α stimulation, Y397-FAK phosphorylation was upregulated in cells of undifferentiation, 24-h preinduction and 5-h induction, while no obvious change was detected in MSCs of 18-h and 48-h maintenance (Fig. 3a and b). SDF-1α treatment also led to a significant increase in Y31-paxillin phosphorylation in all MSCs except cells of 5-h induction and 48-h maintenance. Meanwhile, Y118-paxillin phosphorylation was increased in the undifferentiated and 24-h preinduction MSCs, compared with cells of 5-h induction and 18-h maintenance where the increase was lessened and cells of 48-h maintenance where no change of Y118-paxillin phosphorylation was observed after SDF-1α treatment (Fig. 3a,c and d), demonstrating that the differentiation of MSCs affects greatly the phosphorylation of FAK and paxillin in response to SDF-1α, which might contribute to the assembly of FAs and the chemotactic response of MSCs.

### Dynamics of FAs in MSCs under varying differentiation states during directional migration

**Figure 4:**
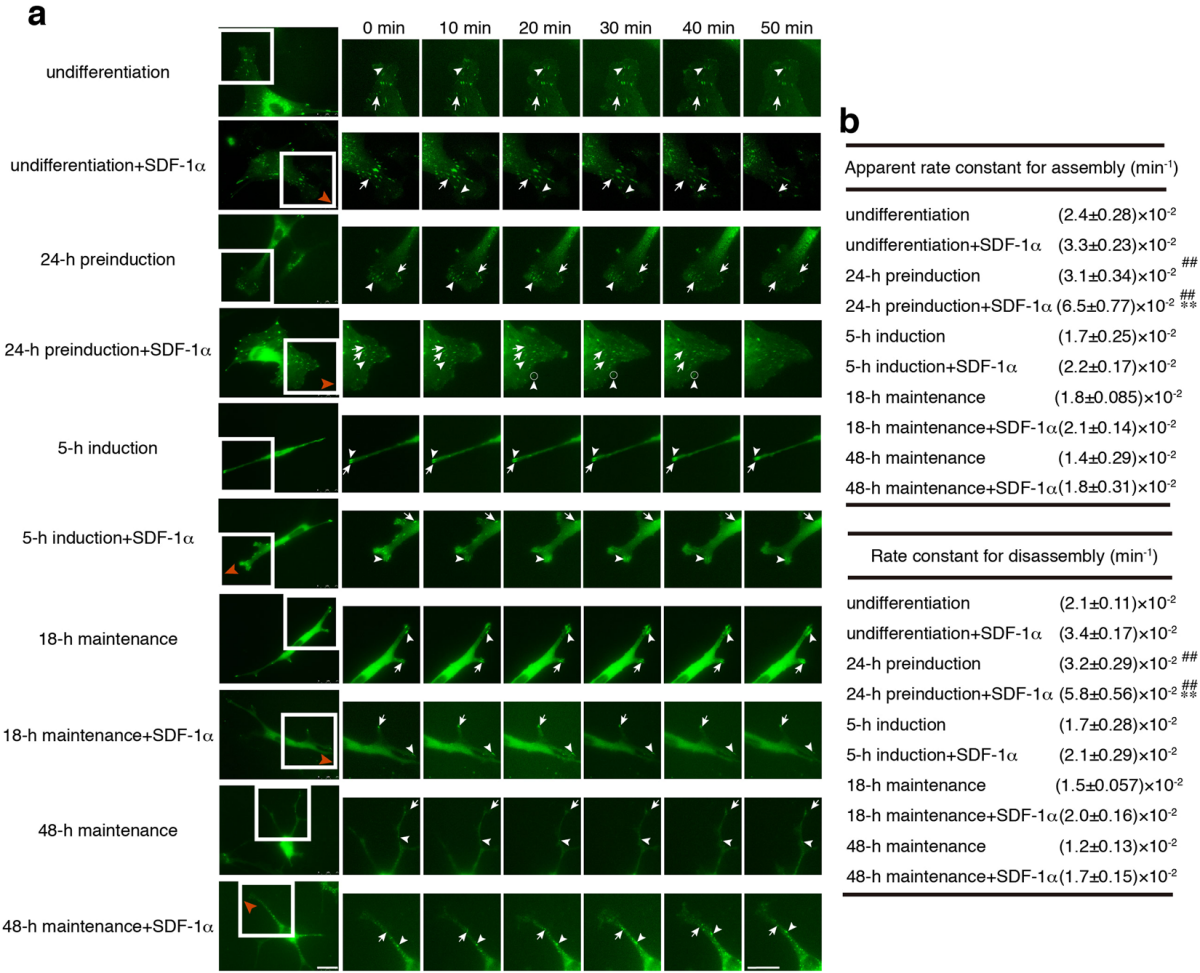
FA turnover in MSCs during chemotactic migration to SDF-1α. (**a**) By using a Dunn chamber and time-lapse video microscopy, time-lapse imaging of pEGFP-paxillin in MSCs were taken every 30 seconds for 1 hours (representative pictures recorded by time-lapse video microscopy at 10-minute intervals for 60 min), showing the differential dynamics of paxillin-containing FAs in cells of varying neural differentiation states (undifferentiation, 24-h preinduction, 5-h induction, 18-h maintenance and 48-h maintenance) with or without the stimulation of SDF-1α. The representative position in the boxed area is enlarged to the right side. Red arrowheads indicate the source of SDF-1α. White arrowheads indicate the assembly, and white arrows indicate the disassembly of FAs. Scale bar: 25 μm. (**b**) Apparent rate constants for assembly and rate constant for disassembly of FAs. Data represent the mean ± SEM from at least three independent experiments. Differences were considered statistically significant when *P < 0.05, **P < 0.01 compared with SDF-1α group of each state and ^#^P < 0.05, ^##^P < 0.01 compared with any other groups under the same treatment.

FAs continuously assemble and disassemble in migrating cells, a dynamic process termed FA turnover^13^. To assess the changes of FAs, we took advantage of the direct-viewing Dunn chamber and time-lapse video microscopy to monitor adhesion dynamics with EGFP-tagged paxillin in MSCs during chemotactic migration to SDF-1α. Given that FAs are much more dynamic at the leading edge of the migrating cells, we thus analyse the turnover of these FAs in MSCs that are exposed to the gradient of SDF-1α in relation to their differentiation states. Time-lapse video analysis revealed that compared with undifferentiated MSCs, cells of 24-h preinduction showed faster FA assembly (arrowhead in Fig. 4a) and disassembly (arrow in Fig. 4a) rates while lower FA turnover was detected in cells of 5-h induction, 18-h maintenance and 48-h maintenance that displayed most stable FAs (representative sequential images shown in Fig. 4a). Upon SDF-1α stimulation, the chemotaxing MSCs, especially cells of undifferentiation and 24-h preinduction, but not cells of 48-h maintenance, displayed a typical polarized morphology with larger persistent lamellipodia at the leading edge and more small FAs with rapid turnover were formed. By following sets of sequential images from time-lapse video (Fig. 4a), we found that FAs assembled and disassembled more rapidly in MSCs exposed to SDF-1α, and that SDF-1α stimulation led to the most obvious reduction in the existing time of FAs in MSCs of 24-h preinduction (Fig. 4a).

To quantitatively analyse the turnover of FAs by live imaging, we calculated the apparent rate constant for assembly and rate constant for disassembly as described in Methods. We found that as compared with undifferentiated MSCs, the rates of FA assembly and disassembly in cells of 24-h preinduction were significantly increased by 29% and 52% respectively, and slightly decreased in other differentiating cells. Upon SDF-1α stimulation, the turnover of FAs was significantly increased in all MSCs and the most significantly increase was observed in cells of 24-h preinduction with approximately 210% increase in the apparent rate constant for assembly (~6.5 × 10^−2^ min^−1^ vs ~3.1 × 10^−2^ min^−1^) and 181% increase in rate constant for disassembly (~5.8 × 10^−2^ min^−1^ vs ~3.2 × 10^−2^ min^−1^), respectively (Fig. 4b). Taken together, we conclude that SDF-1α promotes the turnover of FAs in MSCs that undergo chemotaxis and that the most intense promotion was observed in cells of 24-h preinduction.

### PI3K/Akt and MAPK signallings are implicated in the chemotactic migration and assembly of FAs in SDF-1α-stimulated MSCs

Accumulating evidence implicates PI3K/Akt and MAPK signalling pathways in regulation of cell migration^22, 24, 37^. To further investigate the functional roles of PI3K/Akt and MAPKs (ERK1/2, p38MAPK, and SAPK/JNK) in the chemotactic migration, FA assembly and organisation of F-actin in SDF-1α-stimulated MSCs, cells were pretreated for 30 min with 30 μM LY294002 (PI3K/Akt inhibitor), 50 μM PD98059 (MEK/ERK1/2 inhibitor), 30 μM SB203580 (p38MAPK inhibitor), or 10 μM SP600125 (SAPK/JNK inhibitor), respectively, and the efficiency and speed of migration, the total number and size of FAs as well as the arrangement of F-actin were then examined.

**Figure 5:**
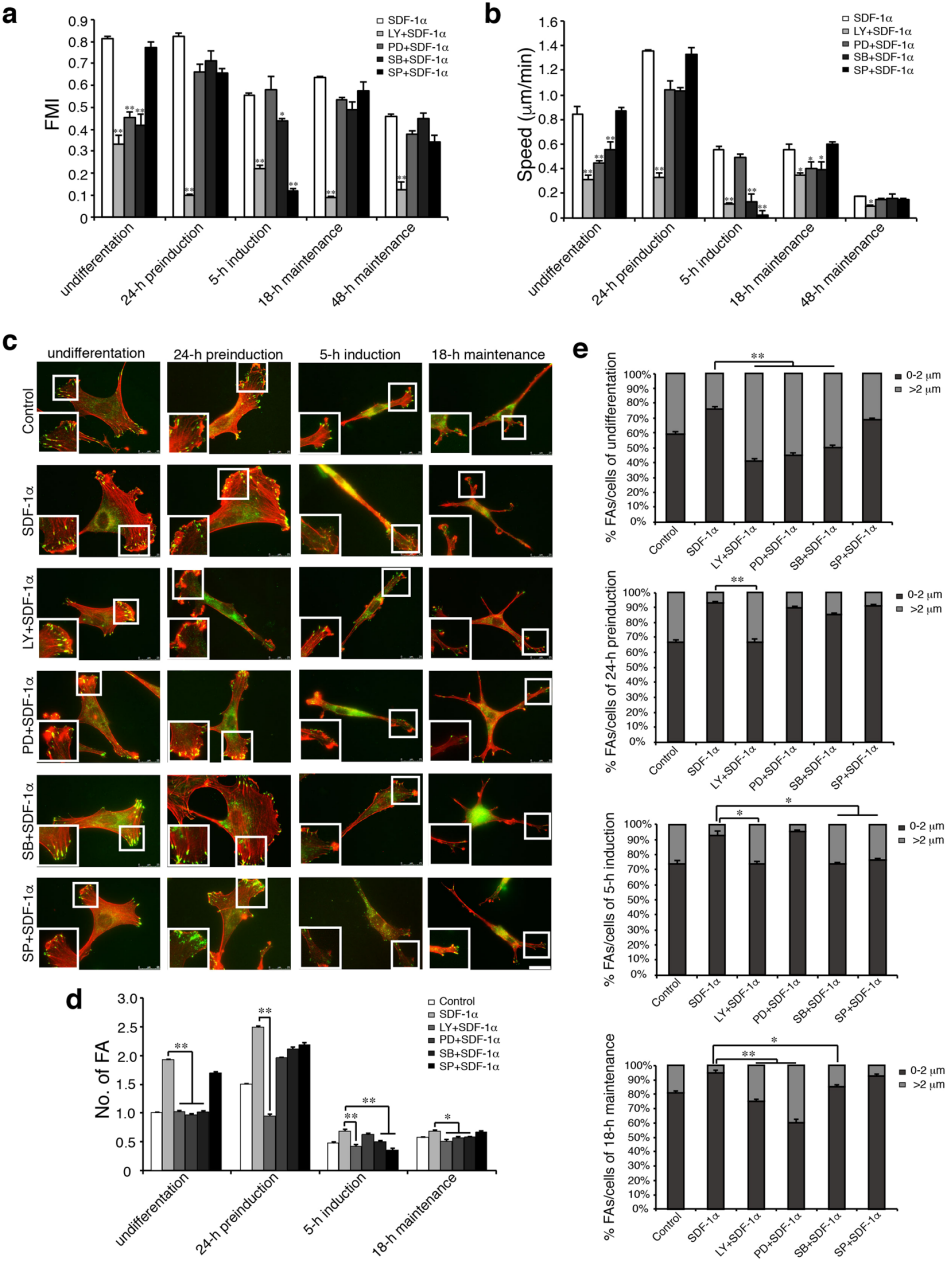
Effects of PI3K/Akt and MAPK signalling pathways on SDF-1α-induced chemotactic migration and FA assembly in MSCs. Cells were treated with LY294002 30 μM, PD98059 50 μM, SB203580 30 μM, or SP600125 10 μM with SDF-1α after serum-starvation. (**a,b**) FMI values and migration speed (μm/min) of MSCs were analyzed at varying differentiation states under the stimulation of SDF-1α and PI3K/Akt and MAPK signaling inhibitors. FMI is the ratio of effective displacement to the total path length. Migration speed was calculated for each time-lapse interval (5 minutes), and the mean speed was derived for 4 hours (>20 cells, n = 3). (**c**) Cells were double stained with paxillin (green). The representative position in the boxed area is enlarged to the bottom left corner. Scale bar: 25 μm. (**d**,**e**) The number and length of FAs (n > 400) in SDF-1α-treated and inhibitor-treated MSCs were measured using the NIH imageJ software. Then, the percentage of FAs (0–2 μm or >2 μm) to the total FAs (% FAs/cell) was calculated (>20 cells). Values were expressed as a percentage of undifferentiated MSCs without SDF-1α. Data represent the mean ± SEM from at least three independent experiments. Differences were considered statistically significant when *P < 0.05, **P < 0.01 compared with SDF-1α group of each state.

Above results indicated that SDF-1α promoted the efficiency and speed of migration in MSCs at varying differentiation states (Fig. 1). To determine whether this effect of SDF-1α treatment is PI3K/Akt and/or MAPK signalling dependent, the outer well was filled with induction medium containing SDF-1α and PI3K/Akt or MAPK signalling inhibitor and the concentric inner well with induction medium containing PI3K/Akt or MAPK signalling inhibitor only, and cells cultured on the coverslip that was inverted onto the chamber were captured every 5 min for 4 h. As shown in Fig. 5a and b, inhibition of PI3K/Akt signalling significantly attenuated SDF-1α-stimulated increase of the migration efficiency and speed in all cells tested, including both the undifferentiated and differentiating MSCs, demonstrating the essential role of PI3K/Akt signalling in the SDF-1α-induced chemotactic migration. By contrast, interference with ERK1/2 signalling decreased the migration efficiency and speed of undifferentiated MSCs and the migration speed of cells of 18-h maintenance, with no effects on cells of other differentiation states, and inactivation of p38MAPK signalling led to a deficit of migration efficiency and speed in cells of undifferentiation and 5-h induction and migration speed in cells of 18-h maintenance, while a reduction of migration efficiency and speed was only observed in SDF-1α-stimulated cells of 5-h induction upon exposure to SAPK/JNK inhibitor.

We then addressed whether PI3K/Akt and/or MAPK signaling are involved in the assembly and turnover of FAs in MSCs upon SDF-1α stimulation. As shown in Fig. 5, inhibition of PI3K/Akt signalling resulted in a significant reduction of the total number of FAs and the small dot-like FAs in both the undifferentiated and differentiating MSCs, suggesting the essential role of PI3K/Akt signalling in SDF-1α-stimulated FA assembly and turnover. Interference with ERK1/2 signalling prevented MSCs of undifferentiation and 18-h maintenance from FA formation and turnover in response to SDF-1α, whereas no effects were observed on cells of 24-h preinduction and 5-h induction. Inhibition of p38MAPK signalling led to a deficit of FA assembly induced by SDF-1α in all cells tested except cells of 24-h preinduction, while FA formation and turnover was reduced only in cells of 5-h induction upon exposure to SAPK/JNK inhibitor. Together, these results demonstrate that PI3K/Akt signalling participates in the regulation of SDF-1α-stimulated chemotactic migration, the assembly and turnover of FAs in the undifferentiated and differentiating MSCs, while the effects of signallings via MAPKs, including ERK1/2, p38MAPK and SAPK/JNK are strictly dependent on the differentiation states of MSCs.

## Discussion

Accumulating evidence indicates that MSCs is a safe candidate and has a great potential to become a viable treatment for neurological diseases^38^. MSCs-derived neuron-like cells have been reported to migrate to lesion site and release growth factors to facilitate nerve restoration^39^. However, not all cells have the same ability of homing into the damaged area due to the possible variant migratory capacity of different subpopulation^40^. It is undoubtedly of great importance to identify the responsive MSCs and to delineate the molecular and cellular mechanisms that govern the directed migration, thereby allowing for optimization of the therapeutic potential of MSCs to be employed for regeneration after injury.

The spatiotemporal control of FAs and actin cytoskeleton play pivotal roles in cell migration^41^. It has been found that cells must balance the assembly and disassembly of FAs for cell relocation and forward progression, during which the number, size, and dynamics of FAs are accurately regulated^42, 43^. In our previous study, we demonstrate that the chemotactic migration of MSCs correlates closely with their differentiation states^24, 26^. To understand the molecular and cellular mechanism, we analysed the dynamics of FAs, organisation of F-actin, and the involvement of related signalling molecules in the migration of these cells in response to SDF-1α, which has been previously demonstrated as a chemoattractant for MSCs^26^. We found that, in line with the close relationship between the chemotactic responses and the differentiation states of MSCs, SDF-1α treatment resulted in a dramatic remodeling of FAs accompanied by F-actin reorganisation in these cells, the degree of which varies considerably, depending on their differentiation states: first, the number and size of FAs and polymerisation degree of F-actin are different in MSCs of various differentiation states; second, SDF-1α-induced assembly of FAs, organisation of F-actin and activation of FA molecules are closely related to the differentiation states of MSCs; third, Dunn chamber assay shows that cells, especially those of 24-h preinduction migrate more efficiently and accordingly, have more significantly increased FA turnover in response to SDF-1α; and finally, the activation of PI3K/Akt signalling is necessary for SDF-1α-induced FA assembly in MSCs, while the effects of ERK1/2, p38MAPK or SAPK/JNK are strictly dependent on the differentiation states of MSCs. All these data demonstrate that the changes in the assembly and turnover of FAs, in response to SDF-1α, correlate closely with the differentiation states of MSCs, which might, in turn, contribute to the varying chemotactic responses of these differentiating cells.

Following the methods provided by Woodbury *et al*., we obtained MSCs at various neural differentiation stages (Fig. 1a). Data from Dunn chamber migration assay by live imaging showed that both the migration speed and migration efficiency of MSCs vary greatly, depending on their neural differentiation states and that MSCs of 24-h preinduction exhibit the strongest chemotactic response to SDF-1α as compared with otherwise differentiating cells (Fig. 1e–g). It has previously been reported that SDF-1α-regulated epithelial ovarian cancer cell invasion is accompanied by the increased expression of CXCR4^29^. Our results showed that MSCs in all differentiation states expressed CXCR4 with the most pronounced expression in MSCs of 5-h induction. However, SDF-1α treatment resulted in a significant increase of CXCR4 expression only in MSCs of 24-h preinduction (Fig. 1h), implying the possible contribution of the increased CXCR4 expression to the chemotactic response of MSCs. Consistently, a most significant increase in the number of FAs, especially the small dot-like FAs (less than 2 μm in length) and the polymerisation of F-actin was observed in cells of 24-h preinduction (Fig. 2). Considering that the large ratio of small FAs was usually found in fast-moving cells^44^, these results suggest that the increased assembly, especially of the small-sized FAs contributes to the enhanced chemotactic response of MSCs. Consistently, cells of 24-h preinduction that exhibit most significantly increase in FA assembly possess the strongest migratory capacity.

It is reported that SDF-1α/CXCR4 axis plays a pivotal role in migration, homing, engraftment and survival of MSCs and recruitment of CXCR4^+^ MSCs to the SDF-1α gradient plays a crucial role in neurological recovery^45^. SDF-1α has been confirmed to provide a clinically viable means to improve the homing of MSCs^46^. Evidence suggests that growth factors enhance cell migration and invasion via activation of FA signalling pathway^47^. Herein, our results provide insight into the underlying mechanisms that the dynamics of FAs and reorganisation of F-actin were changed in MSCs under varying neural differentiation states during chemotactic migration to SDF-1α. The assembly and disassembly of FAs is controlled by protein phosphatases that target adhesion molecules including FAK and paxillin, and these phosphorylation events promote lamellipodium protrusion, FA formation, and cell migration^48^. Studies have shown that cells with mutation of FAK at Y397 fail to undergo FA disassembly, leading to large and stable FAs and decreased migration^49^. Paxillin becomes tyrosine phosphorylated on Y31 and Y118 in a FAK-dependent manner upon the ligation of an integrin to extracellular substrate, and then enhance the migration of cells^50^. Previous studies have shown that stimulation of MSCs with SDF-1α significantly results in the activation of FAK and paxillin, and the rearrangement of F-actin^18^, and that the tyrosine phosphorylation of FAK on Y397 and paxillin on Y31/Y118 participates in the regulation of the assembly and turnover of FAs^13, 36^. In the present study, we found that, as a result of SDF-1α treatment, Y397-FAK and Y31/118-paxillin phosphorylation was significantly increased in MSCs of undifferentiation and 24-h preinduction (Fig. 3), reflecting the stronger chemotactic migration of these cells, while no changes in cells of 48-h maintenance, which showed weak migration (Fig. 1), implying the important role of the increased phosphorylation of these molecules. By contrast, in MSCs of 5-h induction and 18-h maintenance that show relatively much smaller migration speed, variation of FAK and paxillin phosphorylation seems not to be closely related to the migration of these cells, suggesting that changes in the FAK and paxillin phosphorylation might be important for the strong chemotactic responses of MSCs towards SDF-1α. Further studies are required to address the close relationship between the phosphorylation level of these molecules and the chemotactic migration of MSCs.

FA turnover in migrating cells is integral for establishing the polar morphology as well as maintaining cell-matrix contacts. Cell polarity requires strengthening of nascent adhesions or maturation of nascent adhesion complexes to focal adhesion^51^. Our time-lapse video analysis using the direct viewing Dunn chamber revealed that MSCs of 24-h preinduction state had much stronger chemotactic response to SDF-1α than cells in other differentiation states both in migration speed and migration efficiency (Fig. 1). During chemotactic migration to SDF-1α, MSCs displayed a typical polarized morphology with more small FAs clustering, formed persistent lamellipodia at the leading edge (Fig. 4). Moreover, we found that both apparent rate constant for assembly and rate constant for disassembly were significantly increased in chemotaxing MSCs, especially in cells of 24-h preinduction in response to SDF-1α (Fig. 4b), demonstrating that SDF-1α-stimulated migration is accompanied with an apparently increased FA turnover, which usually characterise fast moving cells^42, 52^, and that the most effective chemotactic migration of 24-h preinduction MSCs could be attributable, at least in part, to the most increased FA turnover.

PI3K/Akt and MAPK signalling mediate many events relevant to cell migration, including FA signalling pathway^22, 53^. In turn, FA signalling pathway can also activate PI3K/Akt and MAPK signalling^54, 55^. In the present study, we demonstrate that SDF-1α promoted the assembly and turnover of FAs and that the promotion was closely related to the differentiation states of MSCs. Further, we showed that this effect of SDF-1α treatment is PI3K/Akt and/or MAPK signalling dependent. Moreover, we found that these signalling participate in the regulation of FA assembly and turnover with different degrees that are closely related to the differentiation states of MSCs. As shown in Fig. 5, inhibition of PI3K/Akt signalling significantly attenuated SDF-1α-stimulated increase of FA assembly and small FA formation in all cells tested, demonstrating the essential role of PI3K/Akt signalling in the assembly and turnover of FAs. By contrast, interference with ERK1/2 signalling prevented MSCs of undifferentiation and 18-h maintenance from FA formation and turnover in response to SDF-1α, with no effects on cells of 24-h preinduction and 5-h induction, and inactivation of p38MAPK signalling led to a deficit of FA assembly induced by SDF-1α in all cells tested except cells of 24-h preinduction, while a reduction of FA formation and small FA percentage was only observed in SDF-1α-stimulated cells of 5-h induction upon exposure to SAPK/JNK inhibitor, suggesting that the involvement of signallings via MAPKs, including ERK1/2, p38MAPK or SAPK/JNK in the regulation of FA assembly and turnover are strictly dependent on the differentiation states of MSCs.

**Figure 6:**
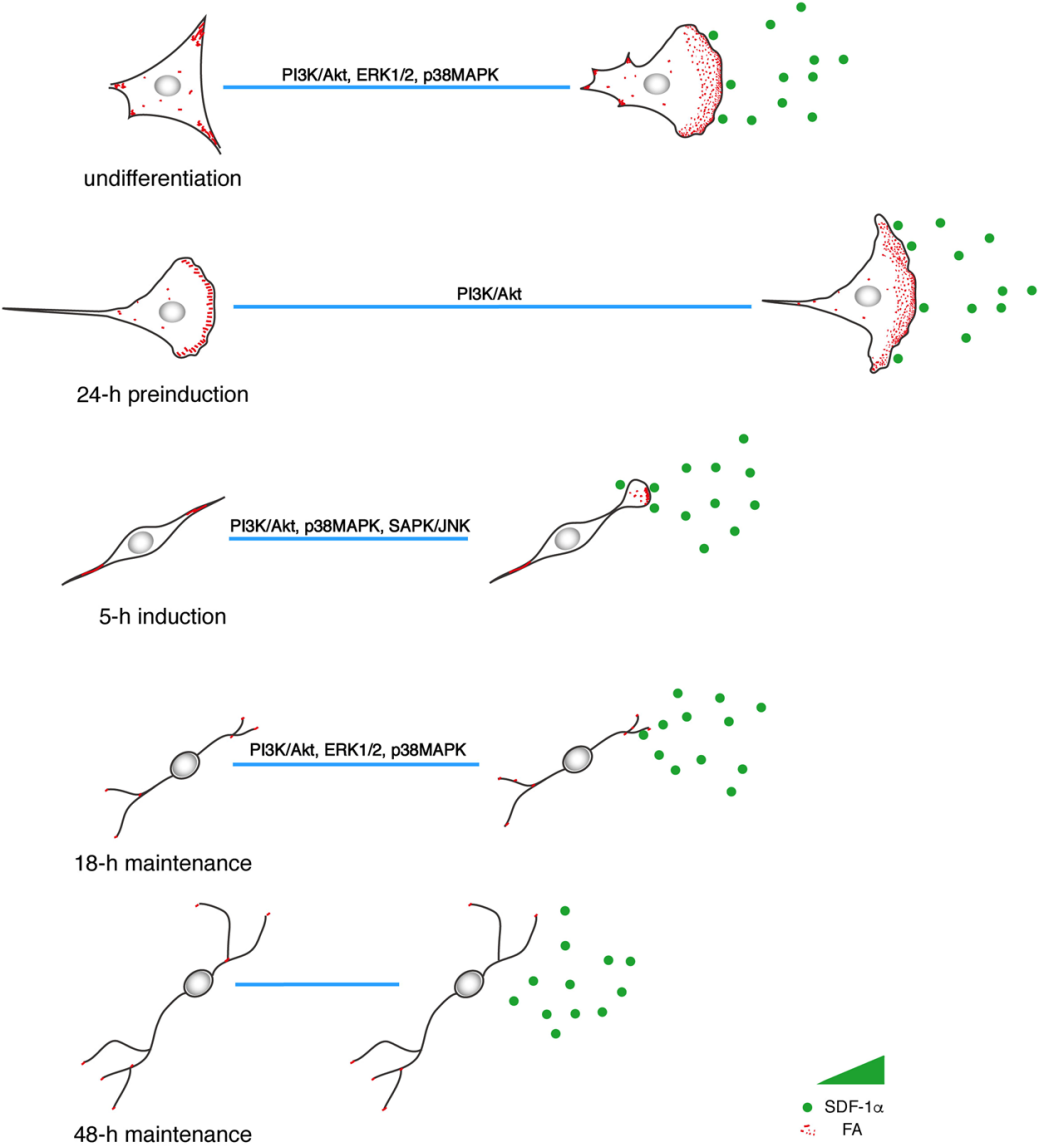
Schematic illustration of the assembly and turnover of FAs in MSCs that undergo chemotaxis towards SDF-1α. MSCs chemotactically migrate towards SDF-1α gradient (depicted as green solid circle) with different migratory capacities that are closely related to the differentiation states (the relative length of the straight blue lines indicate the effective distance cells progressed during the directed migration). SDF-1α induced the formation of lamellipodium at the leading edge with small dot-like FAs (depicted as small red spots; the larger red spots represent the relatively stable FAs), especially in MSCs of undifferentiation and 24-h preinduction. The total number and size of FAs vary considerably, depending on the differentiation states of MSCs. SDF-1α promotes the turnover of FAs in MSCs that undergo chemotaxis and that the most intense promotion was observed in cells of 24-h preinduction that display the strongest positive chemotaxis to SDF-1α. While PI3K/Akt signalling plays a critical role in the assembly of FAs in all the responsive MSCs, albeit with varying effects, the participation of MAPK signallings is strictly dependent on the differentiation states of MSCs: ERK1/2 and p38MAPK involved in MSCs of undifferentiation and 18-h maintenance, p38MAPK and SAPK/JNK involved in MSCs of 5-h induction, no effects in MSCs of 24-h preinduction.

Conclusively, as depicted in Fig. 6, SDF-1α can induce chemotactic migration of both undifferentiated and differentiating MSCs except cells of 48-h maintenance. MSCs in varying differentiation states possess different chemotactic response and cells of 24-h preinduction exhibit the most effective migration. Consistently, the assembly and turnover of FAs correlates closely with the differentiation of MSCs upon SDF-1α treatment and cells of 24-h preinduction show the most increased degree of FA assembly and ratio of small FAs that usually characterise the rapid FA turnover in fast moving cells. While PI3K/Akt signalling plays a critical role in the assembly of FAs in all the responsive MSCs, albeit with varying effects, the participation of MAPK signallings is strictly dependent on the differentiation states of MSCs: ERK1/2 and p38MAPK involved in MSCs of undifferentiation and 18-h maintenance, p38MAPK and SAPK/JNK involved in MSCs of 5-h induction, no effects in MSCs of 24-h preinduction.

All together, results in the present study indicate that FA assembly and turnover, which is accompanied with F-actin reorganisation are closely related to the differentiation states of MSCs that undergo chemotactic migration towards SDF-1α, which might contribute to the different chemotactic responses of the differentiating MSCs, and thus help develop new therapeutic strategy and improve the efficacy of MSCs-based therapy.

## Methods

### Animals

5-week-old Sprague-Dawley rats (100–150 g) were purchased from the Experimental Animal Centre of Soochow University of China. The use of animals was in accordance with Chinese laws, previously approved by Soochow University Veterinary Authority.

### Isolation and culture of MSCs

MSCs were isolated from total bone marrow of femurs and tibias by Percoll gradient centrifugation (1.073 g/ml) and cultured in low-glucose Dulbecco’s modified Eagle’s medium (L-DMEM; Gibco, USA) supplemented with 10% fetal bovine serum (FBS; Gibco, USA), 1% penicillin-streptomycin, and 2 mM L-glutamine (Gibco). Cells were incubated in 95% air and 5% CO_2_ at 37 °C and media was replaced first at 24 h when cells were adherent, elongated, and spindle-shaped in the primary culture of plating; thereafter, the media was changed every 3 days. MSCs at 80%-90% confluence after 12–14 days were harvested by 0.25% trypsin-EDTA solution (Sigma, USA) and passaged at a ratio of 1:2 for expansion purposes. MSCs were used at passages 3–10 in all experiments.

### Preparation of MSCs in varying neural differentiation states

MSCs were seeded at a density of 5 × 10^4^ cells/cm^2^ and neural differentiation was performed with modifications to the procedure described previously (Woodbury *et al*., 2000) when reached approximately 50% confluence. Briefly, MSCs were incubated in the medium consisting of 10% FBS and 10 ng/ml basic fibroblastic growth factor (bFGF; Sigma) in L-DMEM for 24 h (referred to as 24-preinduction), transferred to neural induction medium containing 200 mM butylhydroxy anisole (BHA; Sigma) dissolved in 2% dimethylsulfoxide (DMSO; Sigma) for 5 h (5-h induction), and maintained with H-DMEM containing 1% N2 (Gibco) for 18 and 48 h (18- and 48-h maintenance) respectively.

### Immunocytochemistry

MSCs were seeded on 0.01% poly-L-lysine (PLL; Sigma)-coated glass slides and allowed to extend completely. Cells were fixed in 4% paraformaldehyde for 1 h, then washed three times with PBS (5 min) and incubated with primary antibody for 1.5 h at room temperature or overnight at 4 °C. The primary antibody was diluted in PBS/0.02% NaN_3_/3% bovine serum albumin (BSA)/0.2% Triton X-100 at the following working concentrations: mouse mAb anti-paxillin, 1:140 dilution (BD Biosciences); rabbit mAb anti-Nestin, rabbit mAb anti-β-III tubulin 1:200 dilution (Cell Signaling Technologies). After incubated with primary antibody, cells were washed with PBS three times (5 min), prior to secondary antibody application. FITC-conjugated goat anti-mouse Abs (Proteintech Group, Chicago, IL) were diluted 1:150 in PBS/0.02% NaN_3_/3% BSA and applied to cells for 1 h at room temperature in the dark. Cells were subsequently washed in PBS three times (5 min). For F-actin staining, cells were then incubated with TRITC-phalloidin (1:2,000, Invitrogen) for 40 min, washed with PBS and mounted in 80% glycerol/20% water mix immersion. Fluorescence was examined with Leica DMI 6000 B microscope (Germany). Controls treated with nonspecific mouse IgM, or secondary antibody alone showed no staining.

### Western blot analysis

Cells were exposed to liquid nitrogen and then lysed with protein extraction reagent (25 mM Tris-HCl, pH 7.2, 150 mM NaCl, 1% Trion X-100, 1% sodium deoxycholate, 1 mM EDTA, 0.1% SDS, 1% PMSF, and 1 mM NaVO_3_). Lysates were centrifuged at 12,000 rpm at 4 °C for 15 min to remove cell debris. The cleared supernatants were transferred to fresh tubes and protein concentrations were determined by BCA assay kit (Applygen, Beijing, China). Identical amounts (20–30 μg) of protein lysates were separated using 10% SDS-PAGE gels and transferred to a 0.45mm nitrocellulose membrane (Millipore, Billerica, MA) at a constant 2.5 mA/cm^2^ for 30 min using a Trans-Blot SD Semi-Dry Electrophoretic Transfer Cell (Bio-Rad, Hercules, CA). After blocking with 5% nonfat milk in TBST (100 mM Tris-HCl, pH 7.4, 150 mM NaCl, with 0.1% Tween-20), the membrane was immunoprecipitated with primary Abs for phospho- or nonphospho-protein kinases (rabbit mAb anti-phospho-paxillin (Y31/118), 1:1,000 dilution (Santa Cruze Biotechnology, Inc.); mouse mAb anti-paxillin, 1:140 dilution (BD Biosciences); rabbit mAb anti-phospho-FAK (Y397), rabbit mAb anti-FAK 1:1,000 dilution (Cell Signaling Technologies); mouse mAb anti-β-actin, 1:200 dilution (Boster bio-engineering limited company) overnight at 4 °C. Membranes were then washed three times (10 min), with TBST and incubated for 1 h at room temperature with the appropriate horseradish peroxidase-linked secondary antibody (1:2,000 dilution; Cell Signaling Technologies). Membranes were again washed three times (10 min), and antigen-antibody complexes were visualised by ECL (Biological Industries, BeitHaemek, Israel). The intensity of immunoreactive bands was quantified by gel image analysis software (ImageJ_1.32 J, NIH).

### Cell transfection

MSCs were transfected with DNA using Lipofectamine 2000 (Invitrogen) according to the manufacturer’s instructions. Briefly, MSCs were transfected with 3 μg of EGFP-paxillin plasmids (provided by Clare M. Waterman, Cell Biology and Physiology Centre, National Heart, Lung, and Blood Institute, National Institutes of Health) mixed with 2 μl of Lipofectamine 2000 Transfection Reagent (Invitrogen) in 1 ml serum-free L-DMEM. After transfection for 4 h, cells were incubated 24–48 h in serum-containing medium before observation.

### Dunn chamber migration assay

Chemotaxis of MSCs was directly viewed and recorded in stable concentration gradients of SDF-1α using Dunn chamber (Hawksley, UK), which allowed for generation of a stable chemotactic gradient and observation of cell migration in the context of the gradient^27^. This device is made from a Helber bacteria counting chamber by grinding a circular well in the central platform to leave a 1-mm-wide annular bridge between the inner and the outer wells. Chemoattractants added to the outer well of the device will diffuse across the bridge to the inner blind well of the chamber and form a gradient within 30 min of setting up the chamber. This apparatus allows one to determine the direction of migration in relation to the direction of the gradient. Coverslips with cells transfected by EGFP-target paxillin were inverted onto the chamber, and cell migration or turnover of paxillin was recorded through the annular bridge between the concentric inner and outer wells. In this work, we applied a systematic sampling, and all cells within the migration region of the chamber were recorded and analysed. In chemotaxis experiments, the outer well of the Dunn chamber was filled with induction medium containing SDF-1α and the concentric inner well with only medium. To assess the effect of PI3K/Akt and MAPK signallings on MSCs migration, cells were pretreated for 30 min with 30 μM LY294002 (Promega, Madison, WI), 50 μM PD98059 (Enzo, London, UK), 30 μM SB203580 (Alexis Biochemical, Lausen, Switzerland), or 10 μM SP600125 (Enzo, London, UK), the same concentration of inhibitors was present in both the outer and the inner wells of the Dunn chamber during the migration assays. For migration assay, coverslips with cells were then loaded onto the Dunn chamber and cells were observed every 5 min using 10 × objective by Leica DMI 6000B for a period of 4 h at 37 °C. To determine the efficiency of forward migration during the 4 h recording period, the FMI was calculated as the ratio of forward progress (net distance the cell progressed in the direction of SDF-1α source) to the total path length (total distance the cell traveled through the field)^28^. FMI values were negative when cells moved away from the source of SDF-1α. The cell speed was calculated for each lapse interval recorded during the 4-h period.

### Analysis of FA assembly

The number of FA in MSCs under varying neural differentiation states stimulated with SDF-1α was obtained from an average of 20 cells in each condition from three independent experiments. The number and length of FAs (n > 400) in SDF-1α-stimulated and untreated MSCs were measured using the NIH imageJ software. Then, the percentage of FAs (0–2 μm and >2 μm) to the total FAs (% FAs/cell) was calculated in each condition from three independent experiments.

### Analysis of FA turnover by live-cell imaging

For FA turnover assay, coverslips with cells transfected by EGFP-target paxillin were then loaded onto the Dunn chamber and cells were observed every 30 s using 63 × objective by Leica DMI 6000B for a period of 1 h at 37 °C. The laser was supplied by EL6000 external light source. For EGFP images and FITC images, the 488-nm laser was used, and for TRITC images, the 561-nm laser was used.

FA turnover analysis was as described previously^42, 56^. In brief, the background-subtracted fluorescent intensities of individual EGFP-paxillin inclusive adhesions in cells of varying differentiation states were measured over time using Image Pro-Plus software (Image Pro-Plus 6.0; Media Cybernetics, Silver Spring, MD). To measure the rate constant, the assembly (increasing fluorescence intensity) and disassembly (decreasing fluorescence intensity) period of FAs were plotted on separate semi-logarithmic graphs representing fluorescence intensity ratios in every minute. The apparent rate constant of assembly and disassembly were determined by the calculation of the slopes of linear regression trend lines fitted to the semi-logarithmic plots, which is detailed introduced in Fig. S5. For each rate, measurements were made on at least 10 individual adhesions in 5 separate cells of every differentiation state.

### Real-time quantitative PCR

Total RNA was extracted from MSCs using Trizol reagents (Invitrogen Life Technologies) according to manufacturer’s instructions. 1 μg of total RNA from each sample was reverse-transcribed into cDNA using a reverse transcription kit (Thermo Scientific). Real-time RT-PCR was performed using SsoFast EvaGreen Supermix kit (Bio-rad) with GAPDH as a reference control. Reactions were carried out at 95 °C for 10 min, followed by 40 cycles of 95 °C for 30 s, 60 °C for 30 s, and 72 °C for 30 s. Primers used for real-time RT-PCR were as follows: CXCR4-F, 5′-GAAGTGGGGTCTGGAGACTAT; CXCR-R, 5′-TTGCCGACTATGCCAGTCAAG; GAPDH-F, 5′-TGACAACTTTGGCATCGTGG; GAPDH-R, 5′-TACTTGGCAGGTTTCTCCAGG.

### Statistical analysis

Data are presented as mean ± SEM. Statistical analysis was performed with Student’s *t*-test or ANOVA followed by Bonferroni multiple comparisons test. Differences were considered statistically significant when *P < 0.05, **P < 0.01 compared with SDF-1α group of each state (Student’s *t*-test) and ^#^P < 0.05, ^##^P < 0.01 compared with any other groups under the same treatment (ANOVA).

## Electronic supplementary material

Supplementary Information
