# Supplementary material for: Regulation of focal adhesion turnover in SDF-1α-stimulated migration of mesenchymal stem cells in neural differentiation
Source: Sci Rep. 2017 Aug 30;7:10013. doi: 10.1038/s41598-017-09736-7 (PMC5577153; doi:10.1038/s41598-017-09736-7)
Supplement: Supplementary file 1 — Supplementary Information [file 41598_2017_9736_MOESM1_ESM.pdf]

Supplementary Information

Regulation of focal adhesion turnover in SDF-1α-stimulated migration of mesenchymal stem cells in neural differentiation

Ya'nan Hu, Junhou Lu, Xiaojing Xu, Jingya Lyu and Huanxiang Zhang *

Department of Cell Biology, Jiangsu Key Laboratory of Stem Cell Research, Medical College of Soochow University, Suzhou Industrial Park, Suzhou, China.

*Corresponding author: Huanxiang Zhang, Tel and fax: +86-512/65880277. E-mail: hzhang@suda.edu.cn

Supplementary Figure 1

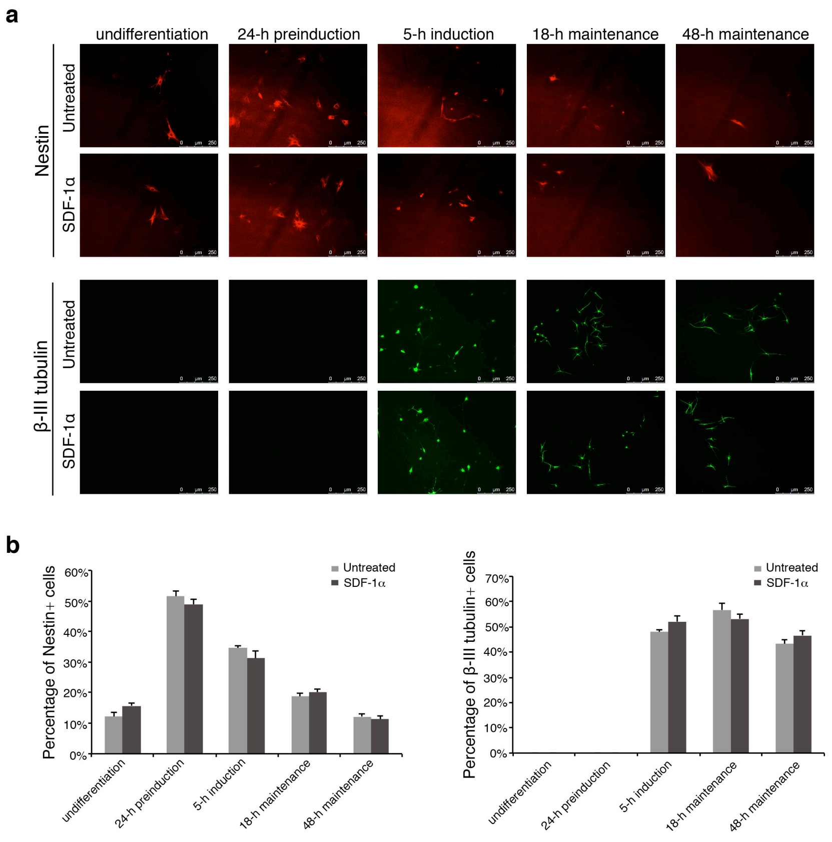

Fig S1. Immunocytochemical characterization of MSCs in neural differentiation

(a) MSCs treated with SDF-1α after serum-starvation were double stained with Nestin (red) and β-III tubulin (green). **(b)** The number of Nestin positive or β-III tubulin positive cells of varying differentiation states were measured using the NIH image J software, and the percentage was calculated (>200 cells) based on the phase contrast images (not shown). Data represent the mean ± SEM from at least three independent experiments.

Supplementary Figure 2

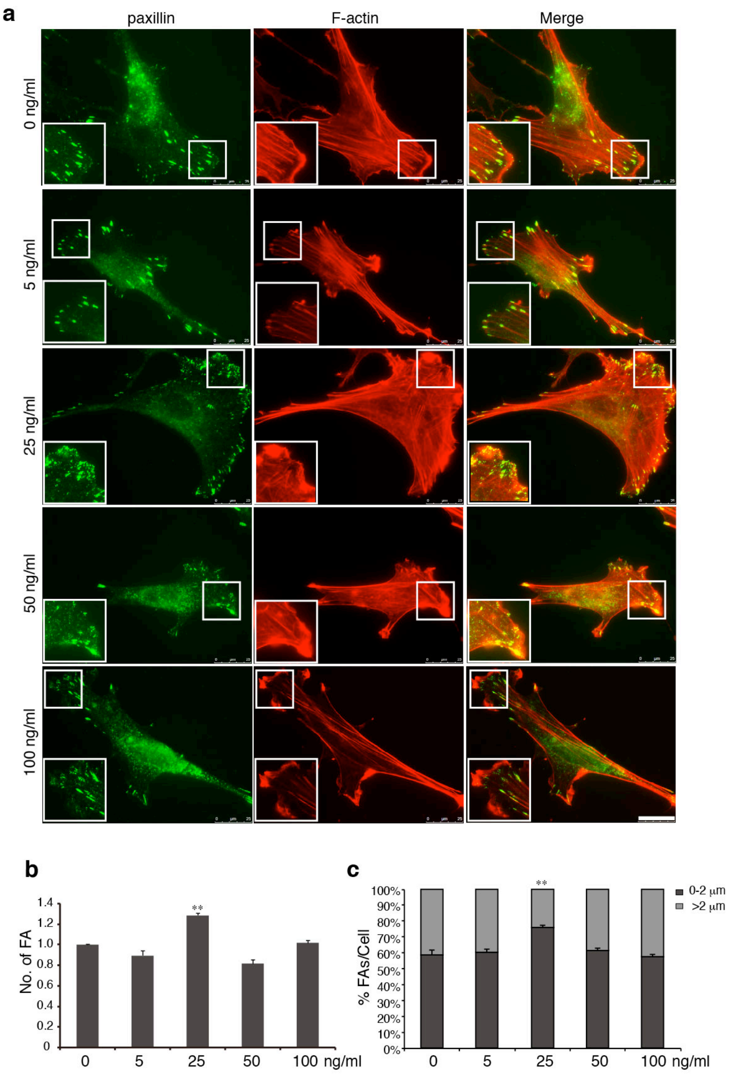

Fig S2. Assembly of FAs, organization of F-actin in MSCs treated with SDF-1α at different concentrations

(**a**) MSCs were treated with SDF-1α at concentrations ranging from 5 ng/ml to 100 ng/ml (5 ng/ml, 25 ng/ml, 50 ng/ml and 100 ng/ml) for 30 min after serum-starvation for 30 min. After stimulation, cells were double stained with paxillin (green) and TRITC-phalloidin (red). The representative position in the boxed area is enlarged to the bottom left corner. Scale bar: 25 μm. (**b**, **c**) The number and length of FAs (n>400) in MSCs under the stimulation of SDF-1α at different concentrations were measured using the NIH image J software. Then, the percentage of FAs (0-2 or >2 μm) to the total FAs (% FAs/cell) was calculated (>20 cells). Values are expressed as a percentage of MSCs without SDF-1α. Data represent the mean ± SEM from at least three independent experiments. *P<0.05, **P<0.01.

Supplementary Figure 3

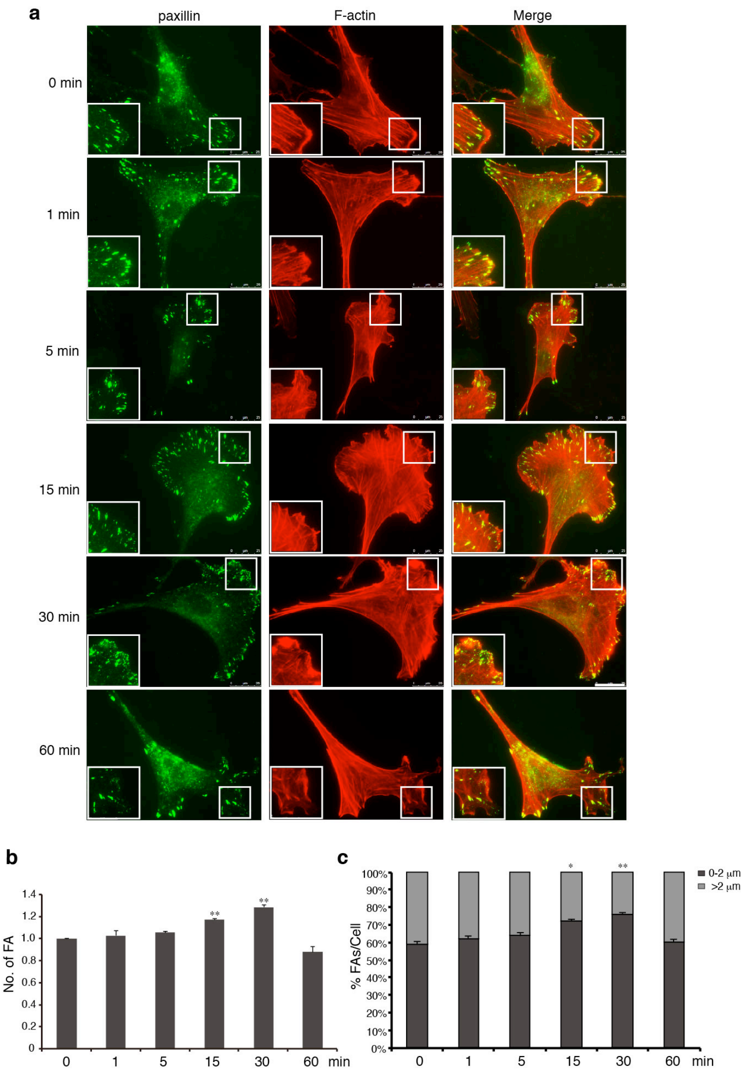

Fig S3. Assembly of FAs, organization of F-actin in MSCs treated with SDF-1α for different time

(**a**) MSCs were treated with SDF-1α at 25 ng/ml for different time (1 min, 5 min, 15 min, 30 min and 60 min) after serum-starvation for 30 min. After stimulation, cells were double stained with paxillin (green) and TRITC-phalloidin (red). The representative position in the boxed area is enlarged to the bottom left corner. Scale bar: 25 μm. (**b**, **c**) The number and length of FAs (n>400) in MSCs under the stimulation of SDF-1α at different time were measured using the NIH image J software. Then, the percentage of FAs (0-2 or >2 μm) to the total FAs (% FAs/cell) was calculated (>20 cells). Values were expressed as a percentage of MSCs without SDF-1α. Data represent the mean ± SEM from at least three independent experiments. *P<0.05, **P<0.01.

Supplementary Figure 4

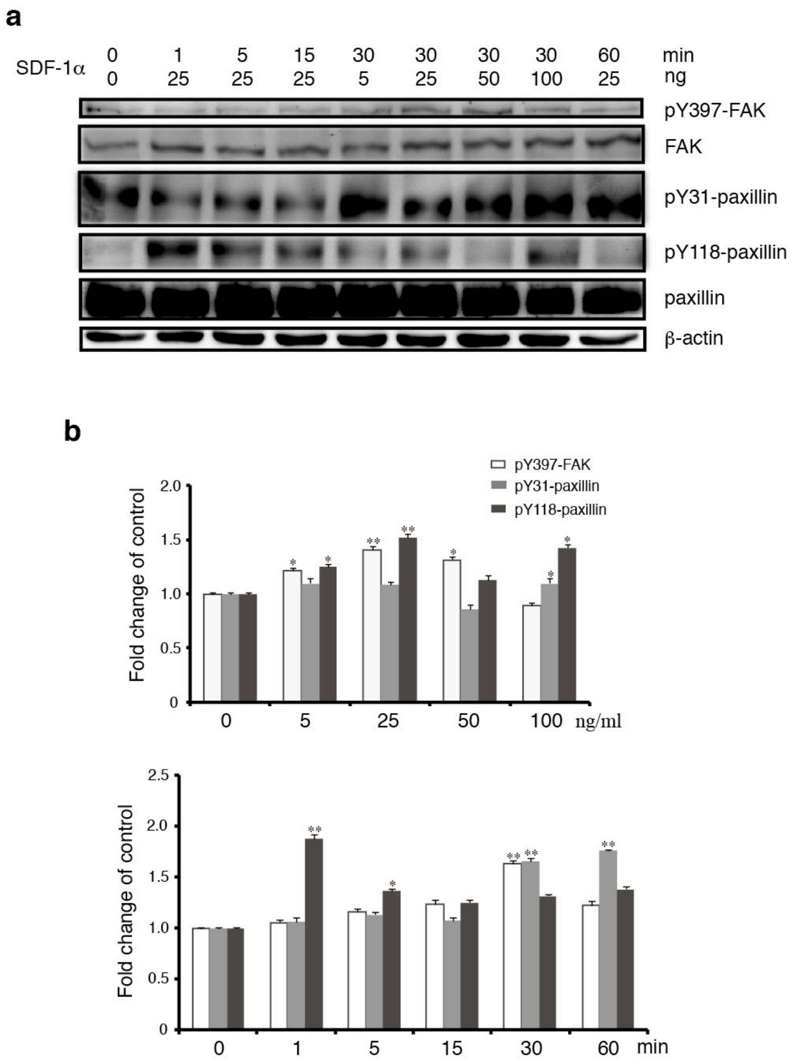

Fig S4. Phosphorylation of FAK and paxillin in SDF-1α-stimulated MSCs

(**a**) MSCs were treated with SDF-1α at concentrations ranging from 5 ng/ml to 100 ng/ml (5 ng/ml, 25 ng/ml, 50 ng/ml and 100 ng/ml) for 30 min or treated with SDF-1α at 25 ng/ml for different time (1 min, 5 min, 15 min, 30 min and 60 min) after serum-starvation for 30 min. After the same stimulation, cells were lysed and immunobloted with primary antibodies for phospho- or nonphospho-protein (FAK and paxillin). (**b**) Levels of Y397-FAK/FAK, Y31-paxillin/paxillin and Y118-paxillin/paxillin were measured by densitometry of immunoreactive bands using ImageJ software. Values were expressed as a percentage of MSCs without SDF-1α. Data represent the mean ± SEM from at least three independent experiments. *P<0.05, **P<0.01.

Supplementary Figure 5

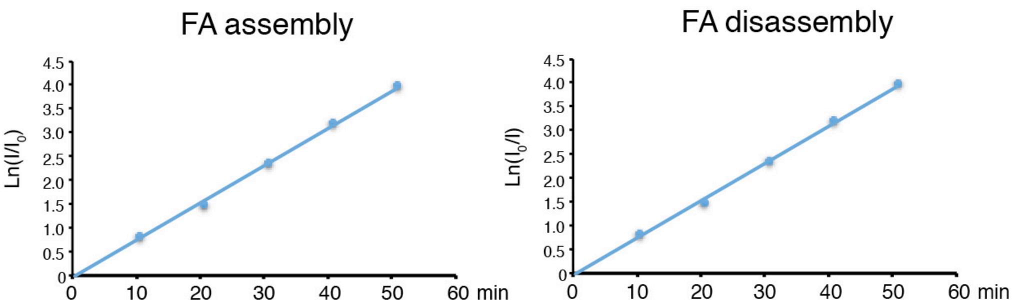

Fig S5. The kinetics of FA assembly and disassembly

The fluorescent intensity of individual adhesions from cells of varying differentiation states expressing EGFP-paxillin was measured over time. I_0 is the initial fluorescent intensity, and I is the fluorescent intensity at the indicated time. The apparent rate constant for formation was determined from the slope of the graph named FA assembly, and the rate constant for disassembly was determined from the slope of the graph named FA disassembly. For each rate constant determination, measurements were made on at least 10 individual adhesions on 5 separate cells of every differentiation state.

Supplementary Figure 6

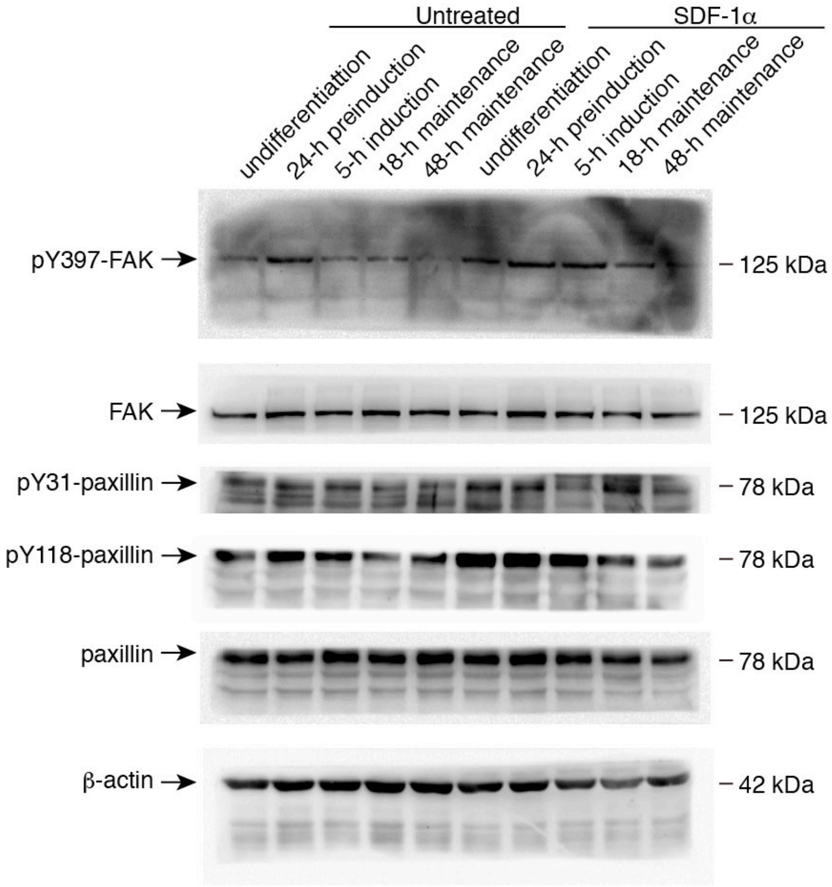

Fig S6. Full-length images of blots presented in the main figures

Full-length blots related to Fig. 3a.
